# Diversity of Transmission Outcomes Following Co-Infection of Sheep with Strains of Bluetongue Virus Serotype 1 and 8

**DOI:** 10.3390/microorganisms8060851

**Published:** 2020-06-05

**Authors:** Eva Veronesi, Karin Darpel, Simon Gubbins, Carrie Batten, Kyriaki Nomikou, Peter Mertens, Simon Carpenter

**Affiliations:** 1 The Pirbright Institute, Pirbright, Surrey GU24 0NF, UK; karin.darpel@pirbright.ac.uk (K.D.); simon.gubbins@pirbright.ac.uk (S.G.); carrie.batten@pirbright.ac.uk (C.B.); Kyriaki.Nomikou@Nottingham.ac.uk (K.N.); Peter.Mertens@Nottingham.ac.uk (P.M.); 2 National Centre for Vector Entomology, Institute of Parasitology, Vetsuisse Faculty, 8057 Zurich, Switzerland; 3 University of Nottingham, Sutton Bonington, Leicestershire LE12 5RD, UK

**Keywords:** Arbovirus, *Culicoides*, Orbivirus, bluetongue, ruminant, infectious disease, transmission

## Abstract

Bluetongue virus (BTV) causes an economically important disease, bluetongue (BT), in susceptible ruminants and is transmitted primarily by species of *Culicoides* biting midges (Diptera: Ceratopogonidae). Since 2006, northern Europe has experienced multiple incursions of BTV through a variety of routes of entry, including major outbreaks of strains of BTV serotype 8 (BTV-8) and BTV serotype 1 (BTV-1), which overlapped in distribution within southern Europe. In this paper, we examined the variation in response to coinfection with strains of BTV-1 and BTV-8 using an in vivo transmission model involving *Culicoides sonorensis*, low passage virus strains, and sheep sourced in the United Kingdom. In the study, four sheep were simultaneously infected using BTV-8 and BTV-1 intrathoracically inoculated *C. sonorensis* and co-infections of all sheep with both strains were established. However, there were significant variations in both the initiation and peak levels of virus RNA detected throughout the experiment, as well as in the infection rates in the *C. sonorensis* that were blood-fed on experimentally infected sheep at peak viremia. This is discussed in relation to the potential for reassortment between these strains in the field and the policy implications for detection of BTV strains.

## 1. Introduction

Bluetongue virus (BTV) is an arboviral pathogen mainly of ruminants and deer that is transmitted primarily by biting midges of the genus *Culicoides* (Diptera: Ceratopogonidae) [1,2]. BTV is a member of the genus *Orbivirus* within the family Reoviridae and its genome comprises 10 linear segments of double-stranded RNA [3], which code for 7 structural and 4/5 non-structural proteins [4,5]. Currently, at least 27 serotypes of BTV have been identified, based on the variability of the virus outer capsid protein VP2 (encoded by genome segment 2), that exhibit only limited levels of cross protection in the ruminant host [6,7,8,9]. In endemic regions, simultaneous infections can frequently occur with multiple BTV strains and/or serotypes in a single host. In some areas, there is also an increased frequency of these events due to the use of live attenuated vaccine strains that can be transmitted by *Culicoides* [10,11,12]. These multiple infections may lead to a high rate of reassortment in the field and recent laboratory-based studies have also demonstrated few apparent constraints on the process of BTV genome segment exchange [13,14].

Since 2006, BTV has caused widespread and economically damaging outbreaks of bluetongue (BT) that have had a significant impact on sheep and cattle production across the Western Palearctic region, through both clinical disease and movement restrictions imposed at a regional and national level to control BTV spread [2,15]. While technologies to produce safe and efficacious inactivated vaccines are available to reduce the impact of BTV outbreaks in northern Europe [16,17], the emergence and re-emergence of strains presents an issue in terms of the proportionate policy response, as the pathogenicity between strains of BTV in different breeds and species of hosts varies significantly [18,19]. This has led to difficulties in defining scenarios under which vaccination should or should not be employed [20,21], both from the perspective of the impact of clinical disease and in reducing the burden of trade restrictions between countries [22,23,24,25].

During the period 2006–2010, the most damaging outbreaks of BT were caused by a BTV serotype 8 (BTV-8) strain, which entered northern Europe directly via an unspecified route from sub-Saharan Africa [26], and a BTV serotype 1 (BTV-1) strain that spread through Morocco into Spain and then into France [1,2]. Both BTV strains were eventually controlled through vaccination campaigns but overlapped in distribution for a significant period of time during 2008–2009 in Spain and France. Anecdotally, these BTV strains differed significantly in their phenotype, most notably in clinical severity (BTV-8 causing significant clinical signs in cattle, which were absent in BTV-1 infection, and BTV-1 having a more severe clinical impact than BTV-8 infection in sheep) and in the occurrence of transplacental transmission in cattle (observed repeatedly in animals infected with BTV-8) [27,28,29,30]. This led to discussion around the policy implications of co-circulating BTV strains in northern Europe, particularly where changes in the spread or pathogenicity of novel emerging strains of BTV had the potential to occur.

A study carried out previously in Belgium examined the co-infection of calves with BTV-1 and BTV-8 strains with a view to explore the potential for competitive exclusion of these strains [31]. Rather than aim to examine the potential for reassortant strains to emerge in northern Europe, this study used serotyping assays to define the likelihood of transmission of BTV-1 and BTV-8 from co-infected calves, as this was a priority for informing predictions of strain spread and subsequent vaccination strategy. While the initial in vivo experiment was confounded by a contaminating strain of BTV serotype 15, a second study using needle-based inoculation demonstrated that BTV-8 replicated to potentially transmissible levels in three calves, while BTV-1 was present only at the limit of detection of the real-time reverse transcriptase polymerase chain reaction (rRT-PCR) assay used to quantify BTV RNA. The quantities of BTV-8 RNA were significantly reduced in these animals when compared to those infected with just the BTV-8 strain.

In our study, we revisited this earlier experiment, following the development of improved in vivo models for the study of BTV infection. We used colony-derived *Culicoides sonorensis*, intrathoracically (IT) inoculated with strains of BTV-1 and BTV-8, to co-infect four sheep with both viral strains simultaneously. We then assessed the development of viral RNA of both strains using an rRT-PCR assay that detected BTV segment 2 (which determines serotype). Following the development of viremia, we then allowed further batches of uninfected *C. sonorensis* to blood feed on co-infected sheep and examined the retention of the two serotypes in the insect host. The aim of the study was to examine the variation in responses to co-infection in both the ruminant and insect host.

## 2. Materials and Methods

### 2.1. Viruses, Vectors, and Hosts Used during Trials

#### 2.1.1. Viruses

Two BTV strains were used throughout the experiments. All isolations and passages were carried out using a cell line established from embryos of *Culicoides sonorensis* in the USA (KC: *C. sonorensis*) [32,33]. Cells were grown in Schneider’s insect cell medium (Lonza, Slough, UK) and supplemented with 1% penicillin/streptomycin (Sigma-Aldrich, Dorset, UK), 1% Amphotericin B (Sigma-Aldrich, Dorset, UK), and 10% Gibco^®^ heat-inactivated fetal calf serum (ThermoFisher Scientific, Waltham, MA, USA) (‘growth media’). A BTV-8 strain (NET2006/06) was isolated from an infected sheep detected in The Netherlands during 2006. Similarly, a BTV-1 strain (MOR2007/01) from an infected sheep detected in Morocco was also isolated on the same cell line. Both viruses were subsequently passaged once on the KC *C. sonorensis* cell line and used as a KC2 passage for vector infection. The titer was quantified using a serial dilution 96-well endpoint titration assay on KC cells incubated for 7 days at 28 °C. Titer of the progeny virus in the cell supernatants was determined using an indirect in-house antigen sandwich enzyme-linked immunosorbent assay (ELISA) to detect the presence of virus in a serially diluted titration of samples [34] and calculated using the method of Spearman and Kärber [35]. *C. sonorensis* were intrathoracically (IT) inoculated using 0.2 µL of tissue culture supernatant of BTV-8 or BTV-1 at a titer of 5.75–6 log_10_ TCID_50_/_mL_ [34].

#### 2.1.2. Vectors

*Culicoides sonorensis* used was the PIRB-s-3 strain, originally derived from the Sorona (AA) line propagated in Denver, Colorado, USA [32]. All *C. sonorensis* utilized in the experiments had emerged from pupae at least four days prior to use.

#### 2.1.3. Hosts

Female adult Dorset Poll sheep (>2 years of age) originating in the UK were used in a group of four animals during the trial, housed in a high containment animal facility at the Pirbright Institute and fed twice a day with grain pellets and with ad libitum access to hay and water throughout the experiment [29]. An uninfected ‘direct contact transmission control’ sheep was housed in the same pen as the treatment animals. All animals were screened for BTV antibodies prior to use using a commercial competitive anti-VP7 antibody ELISA (IDVet, Montpellier, France).

### 2.2. Detection of BTV and Typing

RNA extraction from blood samples containing ethylenediamine tetraacetic acid anticoagulant and rRT-PCR was carried out as described previously [36]. To control for inter-plate variation between independent extractions and rRT-PCR, several replicates (for a 96-well plate, a minimum of 5 replicates) of BTV RNA extracted from positive sheep blood with a known rRT-PCR C_q_ value were included on each extraction/rRT-PCR plate. The sample results of respective plates were only accepted if the positive control samples were within 2 C_q_ values of the previously determined value. Initial detection of BTV RNA was carried out using the rRT-PCR assay based on genome segment 1(Seg-1) detection as previously described and used (termed here as the detection assay) [34,37]. Serotype identification of BTV-1 and BTV-8 strains was conducted directly parallel to the BTV detection assay in potential co-infected samples (sheep blood and orally blood-fed midges) using individual typing rRT-PCR assays based on the BTV genome segment 2 (Seg-2) and quantified by the C_q_ value (termed typing assays) [31]. As previously validated, the separate BTV-1 and BTV-8 typing rRT-PCR assays had the same efficiency and analytical sensitivity [38]. The serotype specificity of the BTV-1/BTV-8 rRT-PCR typing assays used was also confirmed against virus samples of the BTV-1 and BTV-8 strains used in this study as well as additional closely related strains with no evidence of cross reaction.

### 2.3. Infection of Sheep

**Figure 1:**
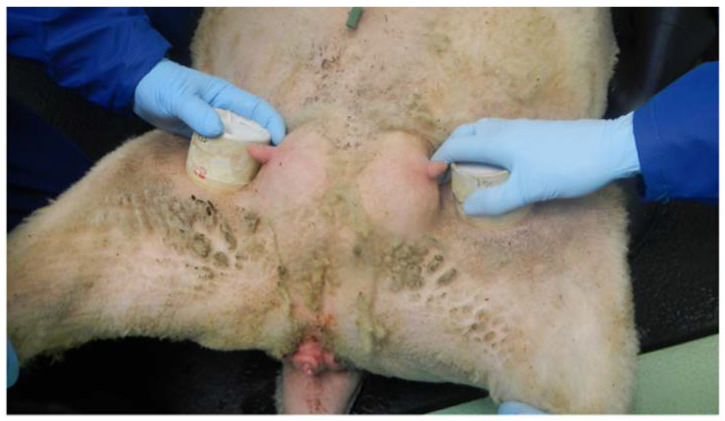
Bluetongue virus (BTV) infection of sheep via blood feeding of infected *Culicoides sonorensis***.** *C. sonorensis* were infected with BTV by IT inoculation and following an incubation period of 5–6 days at 25 °C allowed to feed directly on the inner thigh of a sheep as described in the methodology. Each sheep was simultaneously exposed to one pot containing *C. sonorensis* infected with BTV serotype 1 and one pot containing *C. sonorensis* infected with BTV serotype 8 for 10 min. The sheep was placed onto its rear and held in the sitting position (similar to the position adopted for shearing).

Techniques used to infect *C. sonorensis* with BTV have been described previously [34]. Briefly, over 2000 *C. sonorensis* were intrathoracically (IT) inoculated with 0.2 µL of either the BTV-1 or BTV-8 strain using pulled glass capillary needles (Narishige, London, UK) and a microinjector with a manual syringe driver (Sutter Instruments, California, USA). Inoculation was carried out under light CO_2_ anesthesia and surviving *C. sonorensis* were transferred to specific pill boxes separate for each BTV strain used and incubated for 5 days at 25 °C. A total of 575 *C. sonorensis* survived the incubation period and were used to infect the four sheep (designated sheep 1–4; an unexposed sheep was designated sheep 5 as a transmission control). The same pill boxes were used in which the *C. sonorensis* were stored to reduce the requirement for further anaesthetization and no attempt was made to equalize the numbers exposed to the sheep for each strain to avoid further insect mortality. The four sheep were then restrained and two pill boxes containing BTV-8 NET 2006/06 (pot 1) and BTV-1 MOR 2007/01 (pot 2) IT-inoculated *C. sonorensis* simultaneously placed on both inner thighs for a period of 10 min (Figure 1). Following this exposure, pill boxes were returned to the laboratory, *C. sonorensis* anaesthetized, and the fully disseminated BTV infection rate was quantified in those individual *C. sonorensis* that had taken a blood meal using the same rRT-PCR assay (as serotypes were separated and known), as defined in a previous study [34].

### 2.4. Monitoring of Sheep

Blood samples were taken from the jugular vein on the day before the experiment began (day 0) and then on days 1, 2, 3, 4, 5, 6, 7, 8, 9, 10, 12, 14, and 16 following infection. Clinical signs of disease and rectal temperature were recorded for each animal daily. A postmortem of all sheep was carried out following euthanasia at 16 days post infection (dpi), or when individuals reached a humane endpoint for the protocol. Blood samples taken in the first 10 days post infection were tested for their quantity of viral RNA by rRT-PCR on the same day. Nucleic acid was extracted from 50 µL of the blood sample and the quantity of viral RNA in the sample subsequently assessed by detection and typing rRT-PCR assays. Preliminary data suggested that a blood sample with a rRT-PCR C_q_ value of less than 25 would result in the animal reaching peak viremia within two days.

### 2.5. Clinical Score Calculation

A daily clinical score was recorded for each animal. The score was based on the following 11 criteria: 1. Redness of eyes; 2. Redness of mucosal membranes (oral and nasal); 3. Facial edema; 4. Salivation; 5. Nasal discharge; 6. Cough; and 7. Ulcers (oral and/or nasal) and were all scored daily from 0.5 (very mild) to 3 (severe) (increments of 0.5 were used). 8. Temperature was incorporated into the daily score as follows: The normal body temperature of each individual sheep was determined for at least 3 days prior to infection and the average body temperature was used as the “baseline”. An increase of ≥1 °C from the baseline temperature incurred a clinical score of 1; ≥1.5 °C of 2 and ≥2 °C of 3 for each day of occurrence. 9. A behavior score was included as follows: Apathy and slowness = 1; reluctance to get up unaided and prolonged daily separation from the group = 2; reluctance to get up in response to direct stimulation/body posture indicative of pain = 3 (immediate humane endpoint). 10. A food intake score was determined as follows: Reduced food intake = 1; avoiding concentrate but eating hay = 2; no food intake = 3. The food intake was scored across two daily meals and then averaged. 11. Clinical signs of BTV infection in feet was determined separately for each foot, ranging from 0.5–3 and considering warmth, reddening, lesions, and lameness and subsequently averaged for a daily foot score.

Sheep were euthanized if they reached pre-defined humane endpoints of moderate to high severity limits defined within the study protocol in the ethics project license. These endpoints could be reached either by severity of single behavioral or clinical signs or a combination of several signs of moderate severity on a single day.

### 2.6. Pathology Score Calculation

The total gross pathology score was determined through a combination of a hemorrhage score, an edema score, an ulceration score across a standardized range of selected organs, and a lung lesion score; all criteria were marked from 0.5 (very mild) to 3 (severe).

### 2.7. Infection of Culicoides sonorensis

At peak viremia, pots of approximately 250 adult *C. sonorensis* were exposed to the inner thigh of each sheep. Once returned to the laboratory, blood-fed females were selected under light CO_2_ anesthesia and incubated for 8 days at 25 °C. Those surviving this period were selected under CO_2_ anesthesia and placed individually in racked 96 sample polypropylene tubes (Qiagen, Crawley, UK). A selected number of blood-fed females were also processed immediately following blood feeding without incubation to obtain BTV RNA values of uptake only (D0 midges). Individual *Culicoides* were then homogenized using a 3-mm stainless steel ball bearing (Dejay Distribution Ltd., UK) in 100 µL of Schneider’s media (Sigma Aldrich, Poole, UK), before the addition of a further 900 µL of Schneider’s media. Pools of 400 µL were then created in polypropylene tubes using 50 µL of homogenate from each of 8 individuals and screened using the detection rtRT-PCR assay [37]. Where BTV was detected, individual samples from that pool were retested using the detection rtRT-PCR assay and the BTV-1/BTV-8 typing rtRT-PCR assays, as for sheep blood [38].

### 2.8. Statistical Analysis

Levels of viral RNA (C_q_ values) in sheep determined by the three rtRT-PCRs (i.e., segment-1 (detection), BTV-1, and BTV-8 segment-2 (typing)) were compared using a linear mixed model with C_q_ as the response variable, days post exposure and assay and an interaction between them as fixed effects, and sheep as a random effect. Model selection proceeded by stepwise deletion of non-significant (*p* > 0.05) terms (judged by likelihood ratio tests), starting from a model including all explanatory variables. The proportion of *C. sonorensis* positive for viral RNA of each BTV strain after incubation was analyzed using a binomial family generalized linear model with a logit link function, with the proportion of positive midges as the response variable and the virus strain (BTV-1 or BTV-8) and blood meal C_q_ value as explanatory variables. Model selection proceeded by stepwise deletion of non-significant (*p* > 0.05) terms (judged by likelihood ratio tests), starting from a model including all explanatory variables. All analyses were implemented in R (version 3.6.1) [39].

### 2.9. Ethics Statement

All animal experiments were carried out in accordance with the UK Animal Scientific Procedure Act (ASPA) 1986, which transposes European Directive 2010/63/EU into UK national law. All animal experiments were conducted in compliance with a national project license number PPL 70/7819 granted by the UK Home Office to Dr Karin Darpel from 1st September 2013 to 1st September 2018.

## 3. Results

### 3.1. Infection of Sheep

A total of 575 *C. sonorensis* that survived the extrinsic incubation period following IT inoculation were exposed to the sheep (Table 1; Supplementary Table S1). In total, 100 of the 575 *C. sonorensis* took at least a partial blood meal (17% of those exposed), of which 97 produced C_q_ values (using the detection rRT-PCR assay) indicative of full dissemination (≤ 25) [34]. In total, 57 of these fully infected *C. sonorensis* were infected with BTV-1 and 40 were infected with BTV-8 and the proportions feeding on each sheep are shown in Table 1 (Supplementary Table S1).

### 3.2. Monitoring of Sheep

**Figure 2:**
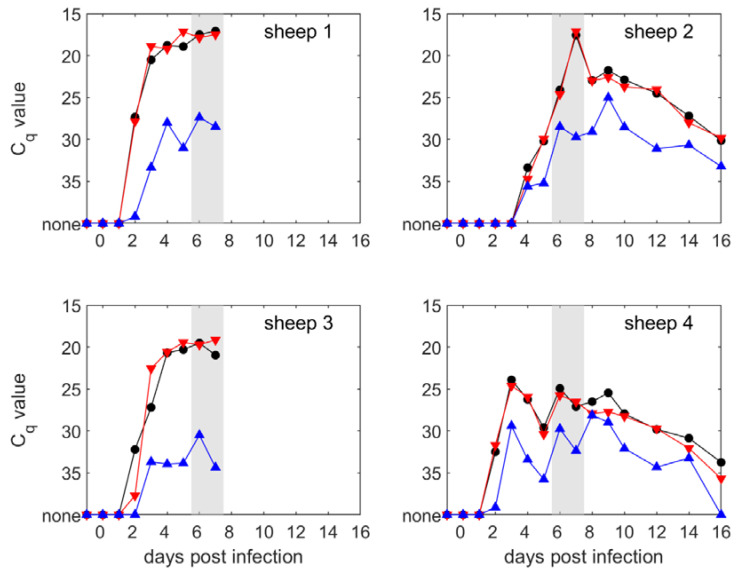
Levels of viremia (measured by rRT-PCR) in sheep co-infected with BTV-1 and BTV-8. Each plot shows the C_q_ values for the detection assay (black circles), the BTV-1 typing assay (red down-triangles), and the BTV-8 typing assay (blue up-triangles). The grey shaded areas indicate when uninfected *Culicoides sonorensis* were fed on the sheep.

Levels of BTV RNA indicative of viremia were demonstrated for all four sheep, although the peak level of viremia in sheep 4 was reduced (Figure 2; Supplementary Table S2). C_q_ values did not differ significantly (*p* = 0.97) between the detection and typing for BTV-1 assays but were significantly (*p* < 0.001) lower for the typing for the BTV-8 assay compared with the other two assays (estimated difference in C_q_ values (95% confidence interval (CI): Typing BTV-8 vs. detection = 5.5 (4.1–7.0); typing BTV-8 vs. typing BTV-1 = 5.4 (3.9–6.8)) (Figure 2). This difference was consistent across time points (i.e., there was no significant (*p* = 0.16) interaction between days post exposure and assay).

In all cases, BTV-1 segment-2 RNA was detected in greater quantities from sheep during the viremic period, including in sheep 2 where a greater number of IT inoculated *C. sonorensis* transmitting BTV-8 (5 fully fed; 5 partially fed) were successfully fed than those transmitting BTV-1 (1 fully fed; 4 partially fed). No BTV RNA was detected in the blood of the transmission control sheep 5, which remained negative for BTV RNA throughout the entire course of the study.

### 3.3. Clinical Scores

**Figure 3:**
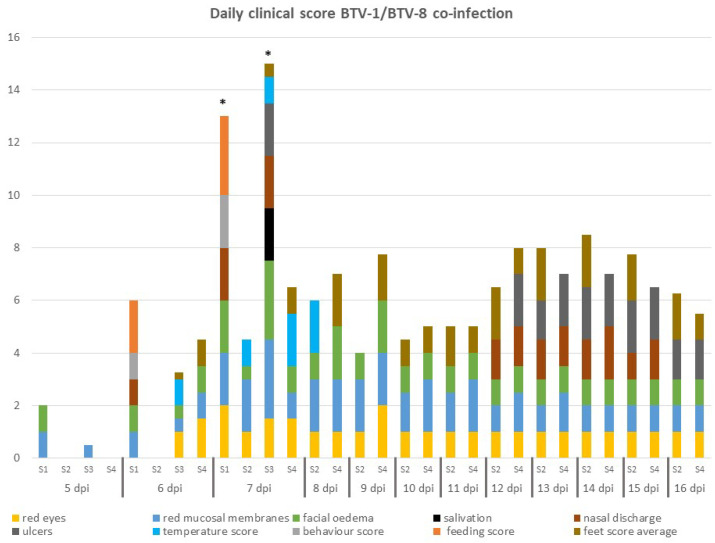
Daily clinical score of sheep co-infected with two BTV strains. The daily cumulative score across 11 clinical criteria are shown for each sheep (S1–S4). Clinical progression was markedly more rapid and severe in sheep 1 and 3 and the total clinical score accumulated on a single day was a clear indicator for overall severity requiring euthanasia (*). Sheep 2 and 4 also developed disease of overall moderate severity, but this was more reflected in the duration and chronic manifestation (ulcers, feet pathology) without affecting any behavior or feeding responses.

**Figure 4:**
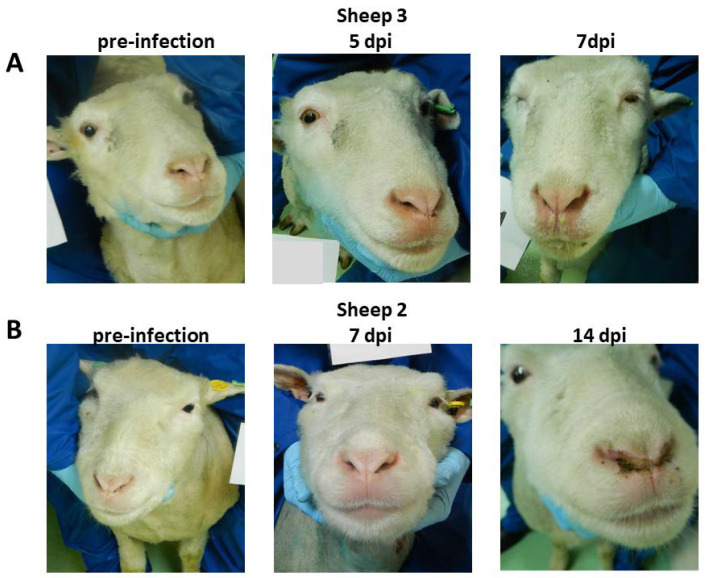
Clinical manifestation of BTV co-infection in Dorset Poll sheep. All four sheep developed typical clinical signs indicative of BTV infection, such as reddening of the eyes and mucosal membranes, facial edema, nasal discharge, and salivation. However, clinical progression was quicker and more pronounced in sheep 1 and 3 (e.g., Panel **A**, sheep 3) and both sheep were euthanized at 7 dpi on reaching the humane endpoints of the study protocol. Sheep 2 and 4 developed moderate clinical BT (e.g., panel **B** sheep 2) and developed more chronic disease features, such as ulcers and petechial bleeding of the nostrils, later in infection (12–16 dpi).

All BTV-infected sheep developed classical clinical signs of BTV infection, including reddening of the eyes and mucosal membranes and facial edema. Clinical signs were first recorded at 5 dpi and daily clinical scores were determined for each individual from this day onwards (Figure 3). Two sheep (sheep 1 and 3) were euthanized on 7 dpi due to apathy and reluctance to get up, as well as cessation of food intake and a more pronounced appearance of several other visible clinical signs (Figure 4). The remaining two sheep, 2 and 4, experienced moderate clinical signs characterized by small petechial ulcerations on the nose/muzzle and redness and inflammation of the feet later in infection (11–16 dpi) (Figure 3 and Figure 4). No clinical signs were recorded in the transmission control sheep 5 at any time during the study.

### 3.4. Pathology Scores

**Figure 5:**
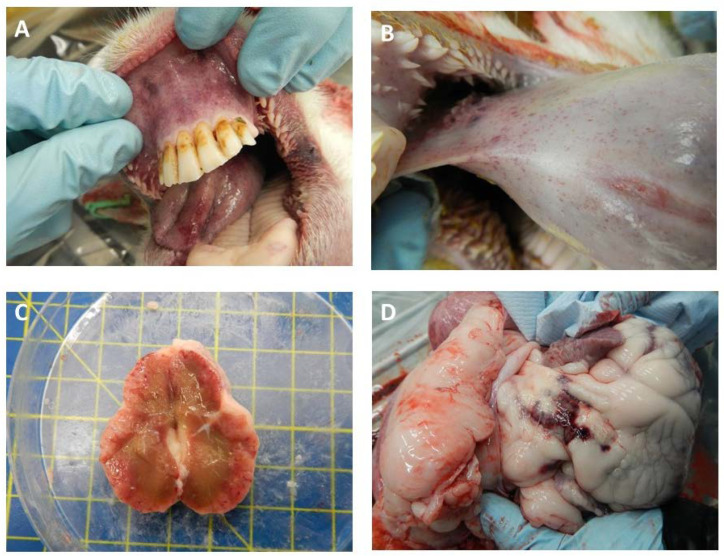
Hemorrhages observed across different organs in BTV co-infected sheep. BTV-specific pathological features observed in sheep 1 and 3 euthanized at day 7 post-infection included hemorrhages, petechial bleeding, and ulceration in the oral mucosa membrane (**A**), tongue (**B**), pre-scapular lymph node (**C**), and pulmonary artery (**D**).

Gross-pathological changes were recorded at necropsy of all four infected sheep. The acute oedemic and hemorrhagic scores were higher in those two sheep euthanized during the acute phase of the disease at 7 dpi (sheep 1 and 3), although hemorrhagic lesions of selected lymph nodes were still present at 16 dpi (Table 2 and Figure 5). Both sheep reaching the study end of 16 dpi (sheep 2 and 4) had evidence of pathological changes, with sheep 2 in particular demonstrating clinical signs associated with BT, such as a hemorrhage of the tongue and pulmonary artery (Table 2 and Figure 5).

### 3.5. Infection of Culicoides sonorensis

A total of 688 *C. sonorensis* were fed on the sheep and then survived the extrinsic incubation period (Table 3). None of the *C. sonorensis* exposed to sheep 1 at 6 dpi imbibed a blood meal, hence no data on viral uptake and/or replication could be obtained for this time point (Table 3). In total, 22 *C. sonorensis* contained BTV RNA at 8 days following feeding on the infected sheep (Table 3; Supplementary Table S3). Feeding on sheep 3 produced 14 infections in *C. sonorensis* (11 with C_q_ ≤ 25; and 3 C_q_ ≥ 25 in the detection assay: 5.6% containing RNA in those exposed); on sheep 4, 2 infections; and on sheep 1, 6 infections (6 C_q_ ≤ 25: 5.1%), while no *C. sonorensis* became infected from feeding on sheep 2. BTV-1 Seg-2 RNA was detected in all positive *C. sonorensis* fed on sheep 3 and 1, and a single individual fed on sheep 1 possessed a mixed BTV-1/BTV-8 infection (typing BTV-1 C_q_: 20.75; typing BTV-8 C_q_: 33.79). BTV-8 Seg-2 RNA only was detected in *C. sonorensis* fed on sheep 4. 

Assuming *C. sonorensis* were infected if they had any C_q_ value, there was no significant difference in the proportion of positive individuals between strains (*p* = 0.27). There was, however, a significant increase in the probability of *C. sonorensis* containing detectable BTV RNA following incubation with a decrease in the C_q_ value of the blood on which it originally fed (odds ratio (OR): 0.77, 95% confidence interval (CI): 0.70–0.86, *p* < 0.001). If blood meal C_q_ was not included as a factor in the analysis, there was a significantly higher proportion of *C. sonorensis* positive for the BTV-1 strain compared with the BTV-8 strain (OR for BTV-8 compared with BTV-1: 0.14, 95% CI: 0.04–0.49, *p* < 0.001). This suggests that the difference in the proportion of positive *C. sonorensis* for each strain is a consequence of higher levels of virus possessing a segment 2 sequence corresponding with BTV-1 than those corresponding to BTV-8 (Table 3). These conclusions remain the same if *C. sonorensis* were assumed to be positive only if they had a C_q_ value < 25.

## 4. Discussion

In this paper, we used a *C. sonorensis* BTV sheep infection model to explore transmission of BTV-1 and BTV-8 strains that co-circulated during the early stages of BTV incursions in northern Europe. In a previous study in which calves were co-infected with BTV-1 and BTV-8 strains using needles, high BTV-8 viremia was detected [31]. In contrast, in our study, using a natural infection and sheep host model resulted in viruses of serotype 1 predominating within all four sheep tested. This even occurred in the case of sheep 2, where more *C. sonorensis* fully infected with BTV-8 were fed to repletion on the sheep than those that were infected with BTV-1. The study also demonstrates that strains of serotype 8 can be recovered in *C. sonorensis* fed on co-infected animals despite relatively low levels of RNA of this serotype being present within the sheep. Finally, one *C. sonorensis* from a total of 22 that developed infections from feeding on viremic sheep developed a mixed infection of BTV-1 and BTV-8, indicating the potential for strains to co-infect the insect host as proposed in studies previously [40].

Intrathoracic infection of *C. sonorensis* using both BTV-1 and BTV-8 consistently led to fully disseminated infections as inferred through the use of rRT-PCR C_q_ values [34]. Successful feeding of infected *C. sonorensis* on the sheep following IT inoculation and incubation is not certain and hence is not standardized. Relatively large numbers of *C. sonorensis* were therefore exposed to the sheep, with numbers successfully feeding ranging from 5 to 23 individuals for infection with either BTV-8 or BTV-1. While the number of *Culicoides* infected with BTV blood feeding on sheep in the field has not been assessed, their populations and subsequent biting rates are among the highest in hematophagous insects [41,42,43,44]. All sheep were successfully simultaneously infected with both strains of BTV through the feeding of *C. sonorensis* with fully disseminated infections, illustrating the high rates of transmission obtained via this route [45,46].

Bluetongue virus RNA was first detected in sheep 1, 3, and 4 on day two post infection (Figure 2). In contrast, RNA was only detected on day 4 post infection in sheep 2 (Figure 2). Similarly, sheep 2 also reached peak BTV RNA levels in blood on day 7 post infection, later than observed in sheep 1 (day 5), sheep 3 (day 5), and sheep 4 (day 3). Sheep 2 was fed on by fewer fully infected *C. sonorensis* (15) than sheep 1 (32), sheep 3 (21), and sheep 4 (32), and this lower rate of inoculation may have led to this delayed onset of RNA detection as demonstrated in a previous study [45].

Co-infection with BTV-1 and BTV-8 was successfully established in all four sheep, despite sheep 2 only being exposed to one *C. sonorensis* infected with BTV-1 taking a full blood meal and four taking partial blood meals. In all four sheep, BTV-1 Seg-2 RNA was detected in greater quantities than BTV-8 Seg-2 RNA at all time points using the BTV typing assays and was closely correlated with Seg-1 RNA detected using the BTV detection assay. There was no visible correlation between the numbers of infected *C. sonorensis* feeding and the peak of RNAemia attained. In addition, the ratio between *C. sonorensis* infected with each serotype feeding on the sheep did not seem to influence the levels of viral RNA for the two strains of BTV.

Divergence in the levels of viral RNA, however, was recorded between sheep 1 and 3 when compared to sheep 2 and 4, with the former experiencing a very high and sustained plateau of BTV-1 Seg-2 RNA levels at < 20 C_q_ and the latter only achieving this for a brief period of 1 day in sheep 2 and not at all in sheep 4. The drivers for differences in the levels of viral RNA were unclear and not correlated either with differences in the number of infected *C. sonorensis* initially imbibing a blood meal from the sheep, or a difference in the ratio between *C. sonorensis* infected with either BTV-1 or BTV-8 taking a blood meal. Both sheep 1 and 3 suffered significantly greater clinical signs than sheep 2 and 4 and this may have been due to the greater quantity of circulating BTV-1 with virus titers ranging 5 to 6 Log_10_TCID_50_ (Table 3). Studies using BTV-1 and BTV-8 strains of the same phylogenetic lineage as used in the current study have reported an earlier onset of clinical disease in BTV-8-infected sheep, but with the greatest severity observed in BTV-1-infected sheep (10–15 dpi). This observation was correlated with an earlier rise in BTV-8 RNA levels but comparable BTV RNA generation detected in sheep with either virus strain at peak infection [30]. Future studies could explore if the initial progression and initiation of viral RNA production (in combination with or independent of peak levels of BTV RNA produced) is at least partially correlated to subsequent clinical disease progression [47].

The infection rates of *C. sonorensis* when fed on the sheep were low and similar to those obtained during previous in vivo studies [45,48] and in epidemiologically relevant *Culicoides* species present in northern Europe fed via membrane-based techniques in Switzerland [49] and pledglet-based methods in the UK [50]. As expected, the likelihood of being able to detect the successful uptake of infectious virus by blood feeding *C. sonorensis* (processed immediately after feeding without any further incubation) was largely correlated with the amount of infectious viral particles circulating in the systemic blood of the sheep on the day of feeding. In our experiments, the exception to this rule were three individuals fed on sheep 2 at 7 dpi (sheep blood C_q_ value of 17.56); otherwise, all individual *C. sonorensis* feeding on a sheep with ≤C_q_21 resulted in a positive rRT-PCR detection of BTV RNA. It is currently unclear why those individuals feeding on sheep 2 did not have detectable amounts of BTV RNA in their blood meal; however, it is important to note that the viral presence in the skin periphery could differ from the level detected systemically in blood samples taken at the jugular vein [51]. Low virus RNA levels were occasionally also detected in individual *C. sonorensis* directly following blood feeding on sheep with less viral RNA in the blood; however, detection was less consistent and at the detection limit of the diagnostic rRT-PCR assay (Table 3 and Supplementary Material Tables S2 and S3).

*Culicoides sonorensis* fed on those sheep with the highest level of virus RNAemia also had the greatest probability of developing infections (Table 3, sheep 1 and 3), and in the majority of individuals, BTV-1 replicated to levels previously indicative of fully transmissible infections (≤C_q_25) [34]. Nonetheless, it is interesting to note that in three individual midges, some degree of viral replication of BTV-8 occurred despite relatively low detected Seg-2 RNA levels (≤28C_q_ in the sheep at the time of feeding), and in the absence of any detectable BTV-8 viral RNA uptake at day 0. Hence, the probability of BTV replication within vector insects following blood feeding on infected sheep does not seem solely explainable by viral levels detected in the systemic sheep blood at the time, although as only segment 2 RNA from BTV-8 was identified it is currently unknown if the virus might have reassorted within the ruminant host.

A key aspect not addressed in the current study is the detection and potential impact of reassortment. Reassortment of BTV can occur when two strains of the virus co-infect a single cell, either in the host or vector. The BTV genome consists of 10 linear segments of double-stranded RNA, and in tissue culture, these segments have been demonstrated to be exchanged with few constraints between European strains of BTV serotype 1 (BTV-1) and BTV serotype 8 (BTV-8) [14]. Analysis of full genome sequences from across European strains of BTV has highlighted that this phenomenon occurs frequently but may also be subject to specific selective pressures that are not fully understood [13,52,53]. Reassortment of BTV strains in the ruminant host were studied in vivo in the USA during the 1980s in both ruminants and vectors [40,54,55]. These experiments highlighted differences in the rate of reassortment between sheep (95% parental strains recovered and 5% reassortant) and a bull (11% parental strains and 89% reassortant). In addition, mixed infection of colony-derived *Culicoides sonorensis* led to proportions of reassortant progeny that varied between 7% and 78% in 8 reassortant-yielding flies. The current study has demonstrated that BTV-1 and BTV-8 duel infections can occur in both the ruminant and insect host and further studies are required to characterize this process.

The policy implications of the current study are significant in highlighting circulation of multiple strains within the ruminant and vector hosts. It demonstrates the importance of serotype determination and full genome characterization where there is doubt regarding the identity of circulating strains. In the case of sheep 4, there was also demonstration that a strain of a serotype that was circulating only at reduced levels of RNA within the ruminant host was transmitted to and replicated within *C. sonorensis*. A key question arising from this observation is whether this represented a reassortant strain and at what point in the cycle of infection this reassortment took place. The study highlights, however, that simple extrapolation from C_q_ values to likely infection of *Culicoides* requires care, particularly in areas where multiple serotypes and strains of BTV co-circulate.

## 5. Conclusions

This experimental study has demonstrated that co-infection of bluetongue virus strains in sheep can lead to a range of outcomes in onwards transmission from sheep by *Culicoides* biting midges. We infected *Culicoides* biting midges with either BTV-1 or BTV-8 by injecting the virus into individuals and then allowing it to replicate within the insect. Then, we used these fully infected *Culicoides* to infect four sheep with both strains simultaneously. Both the clinical signs and the profile and peak level of viral RNA developed in each sheep varied significantly across the sheep alongside clinical disease signs to a greater degree than was expected. We then fed more unexposed *Culicoides* on the sheep to assess infection rates. We found that both strains persisted in the host, in contrast to a previous study, and that infection of *Culicoides* was dependent on being fed when the viruses were at present in the sheep at sufficient levels and a dose-dependent relationship with the specific strain. These results emphasize the importance of using a natural route of infection during BTV transmission experiments.

## Figures and Tables

**Table 1:** Number of intrathoracically (IT) inoculated *Culicoides sonorensis* blood fed on sheep. Each *C. sonorensis* was inoculated with either a bluetongue virus serotype 1 (BTV-1) or BTV-8 strain and then incubated in pill boxes at 25 °C for five days prior to feeding on sheep.

	Sheep Number	Total Inoculated *Culicoides* Exposed	*Culicoides* Fully Engorged	*Culicoides* Partially Engorged	Total Blood Fed (% Total Exposed)	Number ≤ 25 C_q_ * (% Total Blood-Fed on Sheep)
BTV-1 MOR2007/01	1	50	12	5	17 (34)	16 (52)
BTV-8 NET2006/06	1	59	11	4	15 (25)	15 (48)
BTV-1 MOR2007/01	2	71	1	4	5 (7)	5 (33)
BTV-8 NET2006/06	2	68	5	5	10 (15)	10 (67)
BTV-1 MOR2007/01	3	58	14	1	15 (26)	13 (68)
BTV-8 NET2006/06	3	84	5	1	6 (7)	6 (32)
BTV-1 MOR2007/01	4	112	16	7	23 (21)	23 (72)
BTV-8 NET2006/06	4	73	3	6	9 (12)	9 (28)
Total	-	575	67	33	100 (17)	-

* rRT-PCR C_q_ value ≤ 25 indicates *C. sonorensis* containing a fully disseminated infection with bluetongue virus [27].

**Table 2:** Pathology score of all sheep at time point of euthanasia. Sheep 1 and 3 were euthanized as they exceeded clinical limits for the study at day 7 post-infection with bluetongue virus (BTV). Clinical signs exhibited by sheep 2 and 4 were observed to the end of the study (day 16 post infection with BTV).

	Sheep 1	Sheep 2	Sheep 3	Sheep 4
Day of necropsy	7 dpi	End of Study	7 dpi	End of Study
**Clinical Scoring**				
**Edema**	**2**	**0**	**2**	**0**
Face	2	0	2	0
Body	0	0	0	0
Lung	0	0	0	0
Body cavities	0	0	0	0
**Hemorrhage**	**12**	**10**	**14**	**6**
Lymph nodes (Pre-scapular + Pharyngeal + Femoral) + tonsil	1 + 3 + 0 + 3	2 + 2 + 0 + 2	2 + 3 + 0 + 3	1 + 3 + 0 + 2
Mucosal membranes	3	1	3	0
Tongue	2	1	3	0
Muscles and subcutaneous	0	0	0	0
Heart vessels	0	2	0	0
**Ulcer**	**4**	**2**	**1**	**3**
Mouth/Nose	2	2 (mouth and nose 1)	1	2
Feet	2	0	0	1
**Pneumonia**	**0**	**0**	**0**	**0**
**Total Score**	**18**	**12**	**17**	**9**

**Table 3:** *Culicoides sonorensis* blood feeding on sheep co-infected with BTV-1 and BTV-8. *C. sonorensis* were fed at day 6 and day 7 post infection of sheep with bluetongue virus and then stored for 8 days at 25 °C prior to processing.

Sheep (Day)	Sheep BTV Detection Assay C_q_	Sheep BTV-1 Typing Assay C_q_	Sheep BTV-8 Typing Assay C_q_	Sheep Virus ISOLATION (Log_10_TCID_50_)	*Culicoides sonorensis* (*n*) Fed on Sheep	*C. sonorensis* Positive on Detection Assay (% total) Following 8-Day Incubation *	Positive *C. sonorensis* BTV Typing Assays for BTV-1; BTV-8
1 (6)	17.49	17.87	27.37	-	-	-	-
1 (7)	17.07	17.48	28.47	5.75	88	6 (6.8); 6	5 BTV-1; 1 BTV-1 +BTV-8
2 (6)	24.08	24.61	28.47	0	80	0	0
2 (7)	17.56	17.11	29.72	6.0	32	0	0
3 (6)	19.51	19.72	30.49	6.0	112	7 (6.2); 6	All BTV-1
3 (7)	20.97	19.15	34.36	5.0	96	7 (7.3); 5	All BTV-1
4 (6)	24.93	25.75	29.75	0	176	0	0
4 (7)	27.10	26.53	32.36	0	104	2 (1.9); 0	All BTV-8

* rRT-PCR C_q_ value ≤25 indicates *C. sonorensis* fully disseminated infection [27].

## Supplementary Materials

The following are available online at https://www.mdpi.com/2076-2607/8/6/851/s1, Table S1: Levels of bluetongue virus RNA (C_q_) detected in intrathoracically inoculated *Culicoides sonorensis* following incubation for 5 days at 25 °C and exposure to uninfected sheep. Table S2: Levels of bluetongue virus RNA (C_q_) detected in sheep following exposure to *Culicoides sonorensis* intrathoracically inoculated with either a strain of BTV serotype 1 or serotype 8. Table S3: Feeding of uninfected *Culicoides sonorensis* on sheep infected with bluetongue virus. *Culicoides sonorensis* successfully taking a blood meal were processed immediately following feeding and following incubation for 8 days at 25 °C.
